# Features of MRI stromal enhancement with neoadjuvant chemotherapy: a subgroup analysis of the ACRIN 6657/I-SPY TRIAL

**DOI:** 10.1117/1.JMI.5.1.011014

**Published:** 2017-12-23

**Authors:** Adam Olshen, Denise Wolf, Ella F. Jones, David Newitt, Laura J. van ‘t Veer, Christina Yau, Laura Esserman, Julia D. Wulfkuhle, Rosa I. Gallagher, Lisa Singer, Emanuel F. Petricoin, Nola Hylton, Catherine C. Park

**Affiliations:** a University of California San Francisco, Department of Biostatistics and Epidemiology, San Francisco, California, United States; b University of California San Francisco, UCSF Helen Diller Family Comprehensive Cancer Center, San Francisco, California, United States; c University of California San Francisco, Department of Laboratory Medicine, San Francisco, California, United States; d University of California San Francisco, Department of Radiology and Biomedical Imaging, San Francisco, California, United States; e University of California San Francisco, Department of Surgery, San Francisco, California, United States; f George Mason University, Center for Applied Proteomics and Molecular Medicine, Manassas, Virginia, United States; g University of California San Francisco, Department of Radiation Oncology, San Francisco, California, United States

**Keywords:** MRI, breast cancer, neoadjuvant, stroma, enhancement

## Abstract

Although the role of cancer-activated stroma in malignant progression has been well investigated, the influence of an activated stroma in therapy response is not well understood. Using retrospective pilot cohorts, we previously observed that MRI detected stromal contrast enhancement was associated with proximity to the tumor and was predictive for relapse-free survival in patients with breast cancer receiving neoadjuvant chemotherapy. Here, to evaluate the association of stromal contrast enhancement to therapy, we applied an advanced tissue mapping technique to evaluate stromal enhancement patterns within 71 patients enrolled in the I-SPY 1 neoadjuvant breast cancer trial. We correlated MR stromal measurements with stromal protein levels involved in tumor progression processes. We found that stromal percent enhancement values decrease with distance from the tumor edge with the estimated mean change ranging −0.48 to −0.17 (P≤0.001) for time points T2 through T4. While not statistically significant, we found a decreasing trend in global stromal signal enhancement ratio values with the use of chemotherapy. There were no statistically significant differences between MR enhancement measurements and stromal protein levels. Findings from this study indicate that stromal features characterized by MRI are impacted by chemotherapy and may have predictive value in a larger study.

## 1 Introduction

Much like the wound healing process, cancer stroma comprises neovasculature, inflammatory cells, and extracellular matrix components that work cooperatively to support malignant progression.^1^ Through active angiogenesis, stimulated cell proliferation, and the synthesis of connective-tissue, cancer stroma transforms into a highly cellular vascularized tissue. Consequently, cancer stroma has been commonly referred to as activated or reactive stroma.^2^ While its similarity to wound healing is now well understood, the clinically relevant features of activated stroma in treatment and prognosis are unclear. To investigate properties of activated stroma that may play a role in cancer therapy or prognosis, better tools and assessment techniques are necessary. We previously showed that the stroma surrounding breast primary tumors was distinguishable by detectable enhancement patterns using contrast-enhanced breast magnetic resonance imaging (MRI).^3^^,^^4^ Using a tumor proximity mapping technique, stromal enhancement could be related to distance from the tumor edge.^4^ Our previous work suggested that increased peritumoral enhancement was in part attributable to neovasculature^5^ and that chemotherapy-induced changes in global stromal enhancement were associated with disease outcome among a pilot cohort of patients receiving neoadjuvant chemotherapy (NAC).^3^ In this study, we aimed to validate our approach to mapping stromal enhancement and to assess its prognostic value in a prospectively enrolled cohort in the I-SPY 1 TRIAL (investigation of serial studies to predict your therapeutic response with imaging and molecular analysis) with patients receiving NAC and serial MRI scans for breast cancer during treatment. To help identify biological differences among stromal enhancement patterns, we correlated stromal enhancement measured from MRI with stromal proteins that are actively involved in the tumor microenvironment measured from biopsy speciments using reverse-phase proteins microarrays (RPPAs). These proteins are collectively involved in angiogenesis,^6^^–^^8^ wound healing,^6^ inflammation,^9^ and tissue remodeling.^10^^,^^11^ In this study, we test the hypothesis of whether stromal enhancement has a radial dependence on the distance from the tumor edge, and the effect of chemotherapy on global stromal enhancement.

## 2 Materials and Methods

### 2.1 Study Design and Patient Selection

The I-SPY 1 TRIAL was a study of imaging and tissue-based biomarkers for prediction of treatment response and survival. The imaging component of this trial was conducted as a companion study to the American College of Radiology Imaging Network (ACRIN) 6657 and Cancer and Leukemia Group B (CALGB) 150007. Participation in both ACRIN 6657 and CALGB 150007 was required to enroll in the I-SPY 1 TRIAL. All patients gave a signed consent to participate. Eligible patients had histopathologically confirmed breast cancers without evidence of distant metastatic disease. The NAC included an initial four cycles of anthracycline-cyclophosphamide (AC) after which patients either underwent surgery or received four cycles of taxane-based treatment prior to surgery.

### 2.2 MRI Acquisition

The I-SPY 1 TRIAL patients underwent contrast-enhanced MRI at visits before the start of chemotherapy (T1), after one cycle of AC (T2), between AC and taxane-based (T3) regimen, and at the completion of chemotherapy prior to surgery (T4). Images were acquired as previously described.^12^^,^^13^ Briefly, MRI was performed on the tumor bearing breast (ipsilateral) only. Images were acquired on a 1.5-T scanner using a dedicated breast radiofrequency coil. Patients were imaged in the prone position. The MR imaging protocol included a localization acquisition and a T2-weighted sequence, followed by a dynamic contrast-enhanced series. For T2-weighted imaging, a fast spin-echo sequence with fat suppression was used (two-dimensional spin-echo; field of view, 16 to 20 cm; section thickness, 3 mm; fat saturation; echo train length, 8 to 16; one echo; effective echo time, 80 to 140 ms; repetition time, 4000 to 6000 ms). Gadopentetatedimeglumine (Magnevist, Bayer HealthCare) was used as a contrast agent and was injected at a dose of 0.1  mmol/kg of body weight (1.2  mL/s) followed by a 10-mL saline flush. The contrast-enhanced series consisted of a high-resolution (≤1  mm in-plane spatial resolution), three-dimensional (3-D) fast gradient-recalled echo sequence (repetition time ms/echo time ms, ≤20/4.5; flip angle, ≤45  deg; field of view, 16 to 18 cm; minimum matrix, 256×192; 64 sections; section thickness, ≤2.5  mm; voxel size, $29.3\;{\mathrm{mm}}^{3}$). The entire breast was scanned across the sagittal plane with 64 slices for each 2.5 mm thickness over a total scanning time. Imaging time for the T1-weighted sequence was required to be between 4.5 and 5 min, with the data set acquired before injection of the contrast agent ($t_{0}$) and repeated at least two times in the early ($t_{1}$) and late phases ($t_{2}$) after contrast injection. The resulting temporal sampling of the center of k-space for the first contrast-enhanced phase was between 2 min 15 s and 2 min 30 s, providing image contrast most representative of this time point. An interphase delay between the first and the second contrast-enhanced phases was used as needed to result in temporal sampling of the second contrast-enhanced phase between 7 min 15 s and 7 min 45 s.

Despite the long scan duration, the first postcontrast time sample occurred at 2.5 min using the standard k-space sampling, which was close to the effective sampling of 3 min or less that was recommended by the American College of Radiology guideline for breast MRI.^14^

### 2.3 Percent Enhancement and Signal Enhancement Ratio Analysis

**Fig. 1:**
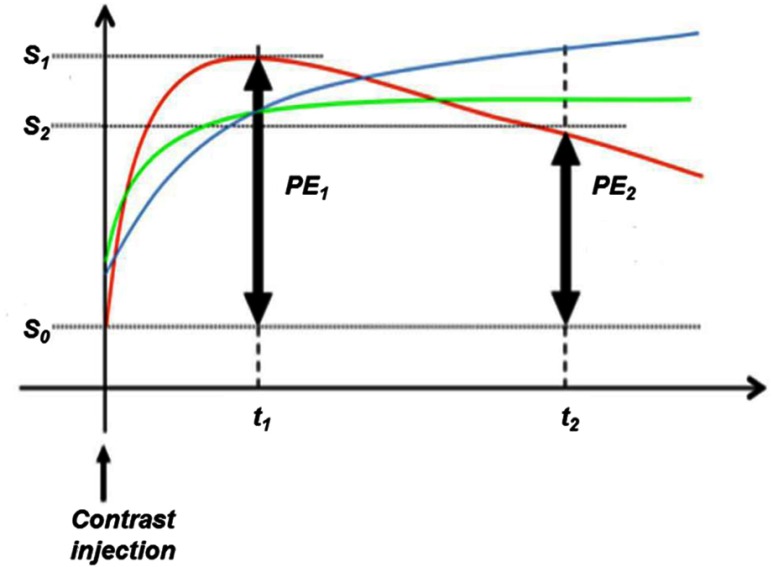
Kinetics of contrast enhancement on breast MRI captured on a signal intensity–time curve. Time points $t_{1}$ and $t_{2}$ are times at which images are acquired after gadolinium contrast injection. $\mathrm{PE}=100\times (S_{1}-S_{0})/S_{0}$ whereas $S_{0}$, $S_{1}$, and $S_{2}$ are signal intensity values in the precontrast ($t_{0}$), early ($t_{1}$), and late ($t_{2}$) postcontrast phases. Signal-enhancement-ratio (SER) is defined as the ratio of PE at $t_{1}$ to PE at $t_{2}={\mathrm{PE}}_{1}/{\mathrm{PE}}_{2}$.

For breast MRI, contrast enhancement kinetics, as captured by the signal intensity–time curves are used to distinguish among malignant (Fig. 1 red curve: rapid rise and signal washout), normal, and benign tissues (Fig. 1 blue and green curves: slow rise, little or no washout). In this signal intensity–time curve, $S_{0}$, $S_{1}$, and $S_{2}$ are the signal intensity values in the precontrast ($t_{0}$), early postcontrast ($t_{1}$), and late postcontrast phases ($t_{2}$), respectively. Percent enhancement [$\mathrm{PE}=100\times (S_{1}-S_{0})/S_{0}$] is calculated for each voxel. Signal enhancement ratio [$\mathrm{SER}=(S_{1}-S_{0})/(S_{2}-S_{0})$ or (${\mathrm{PE}}_{1}/{\mathrm{PE}}_{2}$)] is a method developed to measure contrast enhancement kinetics from high spatial resolution, low 5, contrast-enhanced MR images commonly used for clinical breast MRI.^15^ High SER values consistently identify malignant tissues with a strong signal washout characteristic.^16^

### 2.4 Proximity Mapping of Breast Stroma

**Fig. 2:**
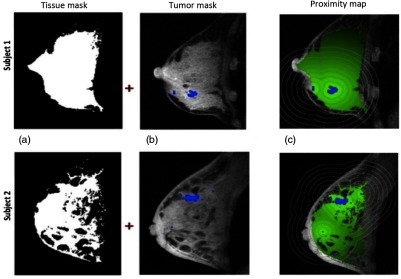
Tumor proximity mapping of breast stroma for two subjects: (a) representative slices of the 3-D FCM derived stroma masks; (b) corresponding slices of the 3-D precontrast T1-weighted MRI with superimposed tumor mask derived from a 70% threshold on the early PE map; (c) T1-weighted image with overlay of tumor proximity, i.e., distance measured from the nearest tumor voxel in the 3-D image. Concentric contours on the proximity map illustrate the 5-mm-thick shells (0 to 5 mm, 5 to 10 mm, etc…) used to define the regions for the calculations in this study.

In this study, contrast-enhanced MRI was used to assess low-level contrast enhancement patterns in the fibroglandular breast tissue outside of the primary tumor. As previously described,^4^ tumor regions on MR images were identified using an enhancement criteria of 70% based on visual agreement with radiological assessments in clinical practice^17^ and were applied to the first postcontrast image.^18^ Normal-appearing stroma surrounding the tumor was defined as fibrogladular tissues and was segmented from adipose tissue using a fuzzy C-means clustering method.^19^ Maps of PE and SER were generated using a customized software program.^18^ The tumor proximity map for normal-appearing breast stroma was generated as shown In Fig. 2 using the 3-D tumor and stromal tissue masks. For each pixel in the stroma, the proximity was defined as the minimum 3-D distance to any tumor pixel. The resulting map is shown as the green overlay in Fig. 2(c). Proximity analysis regions were defined from the proximity map as 5-mm-thick 3-D distance bands from 0 to 40 mm outside of the tumor mask (0 to 5 mm, 5 and 10 mm, etc.), and were applied to the PE and SER maps to calculate the mean PE and SER values within each distance band. Regions closest to the tumor boundary from 0 to 5 mm were considered as tumor periphery.^20^ Global PE and SER values were measured as the average of the mean PE and SER values over 5 to 40 mm, where the tumor periphery was excluded to minimize effects of the tumor masking PE threshold. All subsequent calculations of stromal effects on radial distance, global PE, and SER were focused at regions from 5 to 40 mm from the tumor edge.

### 2.5 Stromal RPPA Analysis

For the analysis of stromal protein levels, breast cancer frozen biopsy specimens in this patient cohort were collected at T2 (N=68) and were subjected to laser capture microdissection (LCM) to enrich for stroma. Stromal populations were isolated (>95% purity) from 8  μm cryosections using an Arcturus Pixcell IIe LCM system (Arcturus, Mountain View, California) as described previously.^21^ Microdissected material was lysed in extraction buffer [tissue protein extraction reagent (ThermoFisher), 2× SDS-PAGE sample buffer (ThermoFisher) mixed 1:1 and 2.5% beta-mercaptoethanol] at a ratio of ∼175 laser pulses/μL of extraction buffer. Samples were heated at 100°C for 5 min, brought to room temperature, briefly centrifuged, and stored at −20°C until printing.

Cell lysates were printed in triplicate spots (∼10  nL per spot) onto nitrocellulose-coated slides (Grace Biolabs, Bend, Oregon) using an Aushon 2470 Arrayer (AushonBiosystems, Billerica, Massachusetts). Standard curves of control cell lysates were also included for quality assurance purposes.^22^ Total protein levels were assessed in each sample by staining with Sypro Ruby Protein Blot Stain (Invitrogen, Carlsbad, California) according to manufacturer’s instructions. Arrays were immunostained with 59 antibodies specific for various stroma-related proteins that were selected based on their involvement in mediating angiogenesis, immune response, inflammation as well as more general pathways such as cell proliferation, motility, and survival (Table 1). All antibodies were validated before use by immunoblotting.^23^ Immunostaining was performed as previously described.^24^ Briefly, each slide was probed with one primary antibody targeting the protein of interest (Table 1). Biotinylated goat anti-rabbit (1:7500, Vector Laboratories Inc., Burlingame, California) and rabbit anti-mouse (1:10, DakoCytomation, Carpinteria, California) IgG were used as secondary antibodies. Signal amplification was performed using a tyramide-based avidin/biotin amplification system (DakoCytomation, Carpinteria, California) followed by streptavidin-conjugated IRDye 680 (LI-COR, Lincoln, Nebraska) for visualization. Images were acquired using a TecanPowerScanner (Tecan, Mannedorf, Switzerland). Antibody staining intensities were quantified using the MicroVigene v3.5.0.0 software package (Vigenetech, Carlisle, Massachusetts). The final results represent negative control-subtracted and total protein-normalized relative intensity values for each endpoint within a given patient sample.^25^^,^^26^

**Table 1:** Primary antibodies used in stromal RPPA analysis.

Endpoint	Dilution	Manufacturer	“Pathway associations”
AKT S473	1:100	Cell signaling	Cell survival
Alpha smooth muscle actin (SMA)	1:50	Abcam	Angiogenesis, myoepthelial cells
ARPC2	1:1000	Abcam	Actin-binding protein, involved in filament nucleation
B-catenin T41/S45	1:50	Cell signaling	Development and tumorigenesis
Caveolin 1	1:100	Santa Cruz	Cell adhesion, apoptosis
Caveolin 1 Y14	1:200	Epitomics	Cell adhesion, apoptosis
CD45	1:200	BD	T and B cell antigen receptor signaling
CD5L	1:50	Sigma	Inflammatory response
Collagen type 1	1:50	Santa Cruz	Extracellular matrix
COX2	1:250	BD	Inflammation
DKK1	1:200	Cell signaling	Regulator of WNT signaling, dysregulation in a variety of cancers
E-cadherin	1:100	Cell signaling	Cell adhesion
Egr1	1:50	Abcam	Transcription factor, neural growth and differentiation
eNOS S113	1:50	Cell signaling	Angiogenesis
eNOS/NOSIII S116	1:500	Upstate	Angiogenesis
ERK1/2 T202/Y204	1:1000	Cell signaling	Cell proliferation
FAK total	1:500	BD	Cell adhesion, survival
FAK Y576/Y577	1:200	Cell signaling	Cell adhesion, survival
FSP (S100A4)	1:200	Millipore	Cell growth and motility, tumor progression marker
ICAM-1	1:200	Cell signaling	Cell adhesion, immune response
IGF-1	1:100	Abcam	Cell proliferation and survival
IGF1R Y1135/Y1136-IR Y1150/Y1151	1:1000	Cell signaling	Cell proliferation and survival
IL-10	1:1000	Abcam	Inflammatory response
IL1B	1:50	Cell signaling	Immune and inflammatory response
IL-6	1:500	BioVision	Immune response
IL8	1:200	Abcam	Promotes angiogenesis
IRAK 1	1:100	Santa Cruz	Cellular stress response, inflammatory response
JAK1 Y1022/Y1023	1:50	Cell signaling	Inflammatory response
JAK2 Y1007/Y1008	1:500	Cell signaling	Inflammatory response
Lamin A, cleaved D230	1:100	Cell signaling	Nuclear envelope protein
LCK Y505	1:50	Biosource	T-cell signaling, mitochondrial apoptosis
LDHA	1:100	Cell signaling	Cell metabolism
MMP-14	1:250	Abcam	Extracellular protease; angiogenesis
MMP2	1:100	NeoMarkers/thermo scientific	Tissue remodeling angiogenesis, tumor invasion
MMP-9	1:1000	Cell signaling	Tissue remodeling angiogenesis, tumor invasion
N-cadherin	1:500	Cell signaling	Cell–cell adhesion; upregulated in cancers
NFkB p65 S536	1:100	Cell signaling	Transcription factor
NGF	1:200	Epitomics	Growth factor signaling
Osteopontin (OPN)	1:100	Assay design	Immune response
p38 MAPK T180/Y182	1:100	Cell signaling	Cell proliferation
PDGFRb total	1:200	Cell signaling	Cell growth and motility
PDGFRb Y751	1:50	Cell signaling	Cell growth and motility
Podoplanin	1:50	Novus	Lymphangiogenesis, stromal factor involved in tumor progression
SERPIN A	1:200	Epitomics	Protease inhibition, inflammatory response
SMAD 1/5/8 SS/SS/SS	1:50	Cell signaling	Cell proliferation, differentiation and apoptosis
SMAD4	1:100	Santa Cruz	Cell proliferation, differentiation and apoptosis
STAT1 Y701	1:500	Upstate	Interferon response
STAT3 Y705	1:200	Upstate	Cytokine and growth factor response
STAT4 Y693	1:100	Cell signaling	Cytokine and growth factor response
STAT5 Y694	1:50	Cell signaling	Cytokine and growth factor response
STAT6 Y641	1:100	Cell signaling	Cytokine and growth factor response
TGF beta 1/3	1:1000	Cell signaling	Cell proliferation and differentiation
TIMP2	1:100	Novus	Tumor angiogenesis and progression
TIMP3	1:50	Cell signaling	Tumor angiogenesis and progression
TWIST 1	1:50	Santa Cruz	EMT
VEGFR2 Y1175	1:50	Cell signaling	Angiogenesis
Vimentin	1:500	Cell signaling	Cellular migration
WNT5ab	1:100	Cell signaling	Tumor development
ZAP70 Y319/SYK Y352	1:100	Cell signaling	Immune response

**Table 2:** Summary of clinical angiographic database of a total of 24 cases.

Characteristics	I-SPY trial evaluable (n=221)	Stromal MRI analyzed (n=71)	*P*-value (MRI versus non-MRI)	3 year-RFS OR (95% CI)	*P*-value (3 year-RFS)	Hazard ratio per unit change (95% CI)	*P*-value (RFS)
Age (years)							
Median (range)	49 (27 to 69)	48 (29 to 69)	0.17	1.0 (0.099, 1.01)	0.93	0.98 (0.94, 1.03)	0.48
Premenopausal	48% (106)	66% (47)	<0.001	0.87 (0.71, 1.09)	0.24	0.66 (0.28, 1.57)	0.36
Race							
Caucasian	75% (165)	79% (56)	0.001	a	0.18		c
African American	19% (42)	8% (6)		1.13 (0.76, 1.69)		c	
Other	6% (14)	13% (9)		0.76 (0.55, 1.05)		c	
Clinical tumor size (cm)							
Median (range)	6.0 (0 to 25)	6.0 (2.5 to 18)	0.51	1.02 (0.99, 1.06)	0.21	1.19 (1.07, 1.33)	0.005
Tumor longest diameter on baseline MRI (cm)							
Median (range)	6.8 (0 to 18.4)	6.4 (2.3 to 11.8)	0.46	1.01 (0.96, 1.06)	0.72	1.20 (0.98, 1.46)	0.08
Clinically node positive at diagnosis	65% (143)	68% (48)	0.65	1.20 (0.95, 1.51)	0.19	2.08 (1.30, 3.33)	0.005
Histologic grade (baseline)							
Low	8% (18)	11% (8)	0.26	a	0.75		0.45
Intermediate	43% (96)	46% (33)		1.12 (0.78, 1.61)		2.51 (0.32, 19.6)	
High	47% (103)	39% (28)		1.15 (0.80, 1.66)		3.10 (0.40, 24.2)	
Indeterminate	2% (4)	3% (2)	b	b	b	b	b
Clinical stage (baseline)							
I	1% (3)	0% (0)	b	b	b	b	
II	47% (104)	52% (37)	0.883	a	0.02		0.001
III	43% (96)	46% (33)		1.28 (1.05, 1.56)		4.94 (1.65, 14.78)	
Inflammatory	8% (17)	1% (1)	b	b	b	b	b
Hormone receptors (baseline)							
HR-positive (ER or PR)	60% (131)	56% (40)	0.55	0.88 (0.71, 1.08)	0.21	0.72 (0.30, 1.69)	0.45
HER2-positive	30% (67)	35% (25)	0.35	1.12 (0.90, 1.39)	0.31	2.22 (0.94, 5.22)	0.07
HR-negative/HER2 negative (triple negative)	24% (53)	24% (17)	1	1.06 (0.83, 1.35)	0.63	1.05 (0.38, 2.87)	0.92
HR-positive/HER2-negative	44% (96)	41% (29)	0.56	0.86 (0.69, 1.06)	0.15	0.40 (0.15, 1.09)	0.06
Chemotherapy response							
RCB0/I	37% (74)	48% (31)	0.028	0.86 (0.69, 1.07)	0.18	0.50 (0.19, 1.32)	0.15
RCBII/III	63% (127)	52% (33)					
							

a The referent group.

b Removed from analysis due to low count.

c Missing due to lack of convergence of the Cox model.

### 2.6 Statistical Analysis

Patient characteristics were compared between I-SPY 1 TRIAL subjects with and without MR stromal analysis using Wilcoxon rank-sum test or Fisher’s exact test as appropriate. Similar comparisons were made between subjects who had 3-year recurrence free survival (RFS) and those who did not. A linear mixed effect model was utilized to assess the PE and SER radial trend in the range of 5 to 40 mm from the tumor edge.^4^ A subject-specific random slope and intercept was included in this model. The variable 3-year RFS and its interaction with distance from the tumor border was included in an additional model. For comparisons with RFS as a censored survival variable, the univariable Cox model was used and testing was performed with the likelihood ratio test. Global PE was evaluated between times utilizing univariable regression. Statistical tests with p-values less than 0.05 were considered significant. Linear regression was used to correlate MR stromal enhancement measurements (global PE and SER) with levels of stromal proteins. All statistical analyses were performed using the R statistical software language.^27^

## 3 Results

### 3.1 Patient and Tumor Characteristics

**Fig. 3:**
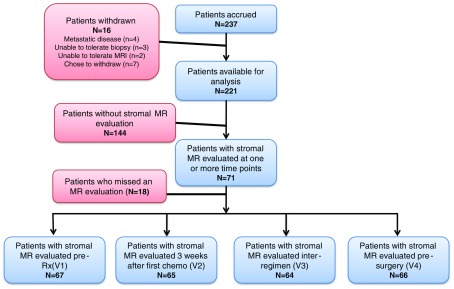
Consort diagram for the study. Patients were accrued to ISPY 1 (n=237). Of these, 221 were available for analysis, and 71 patients had MRIs that were assessable for stromal enhancement. The study protocol was to obtain four MRIs for each patient at V1 (prior to chemo); V2 (after first chemocycle); V3 (between AC and T chemotherapy); V4 (prior to surgery).

As shown in the consort diagram (Fig. 3), there were 221 evaluable patients enrolled in the I-SPY 1 TRIAL. Seventy-one patients had stromal MR evaluation and 144 did not. Characteristics of these patients (total I-SPY 1 versus stromal MRI evaluated) are shown in Table 2. The two groups were similar in age, tumor size, nodal status, subtype, and regimen received. However, notable differences were found in percentage of premenopausal (66% versus 48%), race (relatively fewer African American: 8% versus 19%, and other: 13% versus 6%), and chemotherapy response [residual cancer burden (RCB) class^28^ 0/I: 48% versus 37%; RCB class II/III: 46% versus 63%]. For the analysis of stromal protein levels, breast cancer biopsies were available for analysis at T2 in 68 patients. Measurements of both MR enhancement and stromal protein levels were obtained in 18 patients at T2.

Our survival endpoint was RFS, both at 3 years and as a censored variable. Of the 71 patients with MRI stromal evaluation, 17 patients had recurred after 3 years and 51 had not (three were missing due to insufficient follow up). Univariable Cox proportional hazards analysis showed that clinical stage was significantly associated with RFS (P=0.001). Nodal status [hazard ratio (HR)=2.08, 95% CI (1.30 to 3.33), P=0.005] and tumor size [HR=1.19, 95% CI (1.07 to 1.33), P=0.005] were also significantly associated with RFS. Prediction of RFS by other known prognostic factors including age and chemoresponse were not statistically significant: details of the estimated relationships between these patient tumor characteristics and RFS in terms of hazard ratios, confidence intervals, and p-values can be found in Table 2.

### 3.2 Stromal Enhancement Patterns

As described above, we developed the tumor proximity technique to map the stromal enhancement at incremental distances from the primary tumor edge as visualized on MRI. Using retrospective pilot cohorts, we previously showed that MR PE and SER had higher stromal contrast enhancement patterns in patients with invasive breast cancer.^3^^,^^4^ Here, we validated our previous findings using the ISPY 1 TRIAL cohort and investigated the association of stromal enhancement patterns with treatment.

#### 3.2.1 Radial dependence and interaction with RFS

**Fig. 4:**
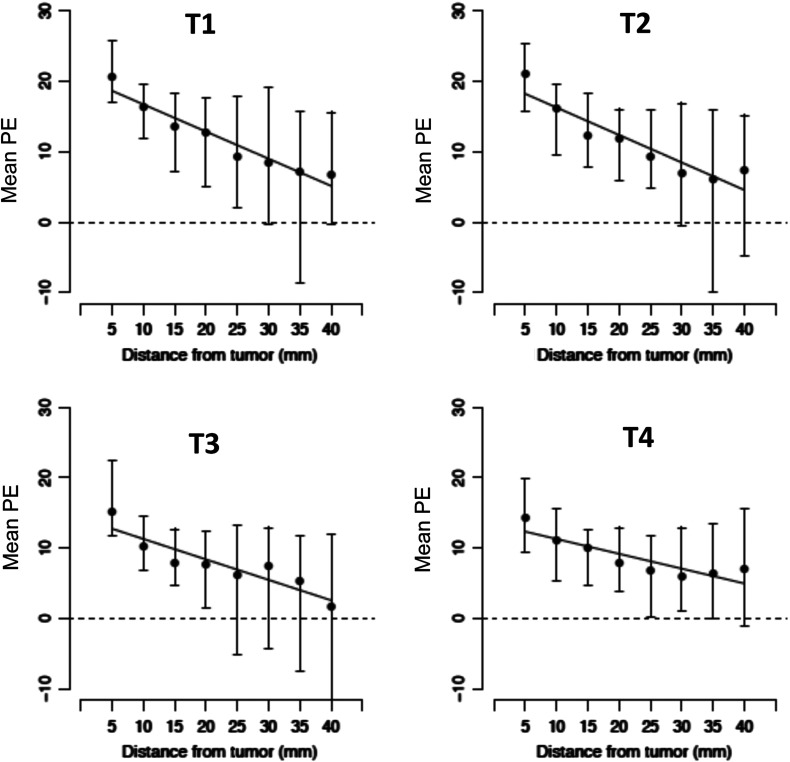
Stromal PE decreases with distance from the tumor edge on average for all time points. The decrease was significant only for time points T2 to T4 (p<0.05 for each).

As shown in Fig. 4, PE values in the I-SPY 1 dataset decreased continuously with distance from the tumor edge: from proximal tissue (within 5 to 10 mm) to distal tissue (35 to 40 mm). Notably, the decreases in PE with distance from the tumor edge remained consistent through all the study time points, T1 to T4 (Fig. 4).

Using the linear mixed effects model, the estimated mean change in enhancement as a function of distance from the tumor edge was calculated for the entire cohort (N=71), Table 3. The estimated mean change in enhancement signal per mm was −0.33 for T1 (95% CI: −0.68, 0.02), −0.38 for T2 (95% CI: −0.55, −0.20), −0.48 for T3 (95% CI: −0.64, −0.28), and −0.17 for T4 (95% CI: −0.26, −0.07). As shown, the corresponding p-values for T1 to T4 were 0.06, <0.001, <0.001, and 0.001, respectively, showing that PE decreases with distance from the tumor edge. All mean changes in PE values were significant except for T1 (P=0.06).

**Table 3:** Change of stromal PE values per mm from the tumor edge at time points before the start of chemotherapy (T1), after one cycle of AC (T2), between AC and taxane-based (T3) regimen, and at the completion of chemotherapy prior to surgery (T4).

	Estimated mean change (per mm)	95% CI	P-value
T1	−0.33	−0.68, 0.02	0.06
T2	−0.38	−0.55, −0.20	<0.001
T3	−0.48	−0.64, −0.28	<0.001
T4	−0.17	−0.26, −0.07	0.001

Next, 3-year RFS, recurrent (N=17) versus nonrecurrent (N=51) was added to the mixed model. The same decreasing radial trend was found in both groups as shown in Table 4. Significant changes were observed in the nonrecurrent patients with estimated mean change per mm of −0.36 for T2 (95% CI: −0.57, −0.15; P<0.001), −0.51 for T3 (95% CI: −0.71, −0.30; P<0.001), and −0.17 for T4 (95% CI: −0.29, −0.05; P=0.005). For the recurrent group, significance change was found only in T2 (estimated mean change permm=−0.39, 95% CI: −0.75, −0.02; P=0.04), but the lack of significance in the recurrent group may be due to the small sample size. The estimated mean difference in distance effects between nonrecurrent and recurrent patients from time points T1 to T4 were −0.01, 0.03, −0.28, and −0.01, suggesting that the decreasing rates of PE from the tumor edge in these groups were similar with the exception of T3. However, limited numbers preclude making a more definitive statement.

**Table 4:** Change in stromal PE values per mm from the tumor edge for recurrent and nonrecurrent patients. Significant changes were found for T2, T3, and T4 in the nonrecurrent patients.

	Recurrent			Nonrecurrent		
	Estimated mean change (per mm)	95% CI	P-value	Estimated mean change (per mm)	95% CI	P-value
T1	−0.3	(−1.02, 0.42)	0.42	−0.31	(−0.73, 0.11)	0.15
T2	−0.39	(−0.75, −0.02)	0.04	−0.36	(−0.57, −0.15)	<0.001
T3	−0.48	(−0.59, 0.14)	0.23	−0.51	(−0.71, −0.30)	<0.001
T4	−0.16	(−0.37, 0.06)	0.15	−0.17	(−0.29, −0.05)	0.005

We also examined changes in PE between adjacent times (T1 versus T2, T2 versus T3, T3 versus T4 in the recurrent and nonrecurrent groups) at the tumor periphery within the nonrecurrent and recurrent groups. In general, the PE signal was lower in the later time point, with the only exception being between T1 and T2 in the nonrecurrent group. The difference was significant only between T2 and T3 in the recurrent group. Here the estimated (pseudo-) median difference was −3.23 per mm (95% CI −6.17, −0.80; Wilcoxon signed-rank test P=0.01).

#### 3.2.2 SER pattern from the tumor edge and interaction with RFS

**Fig. 5:**
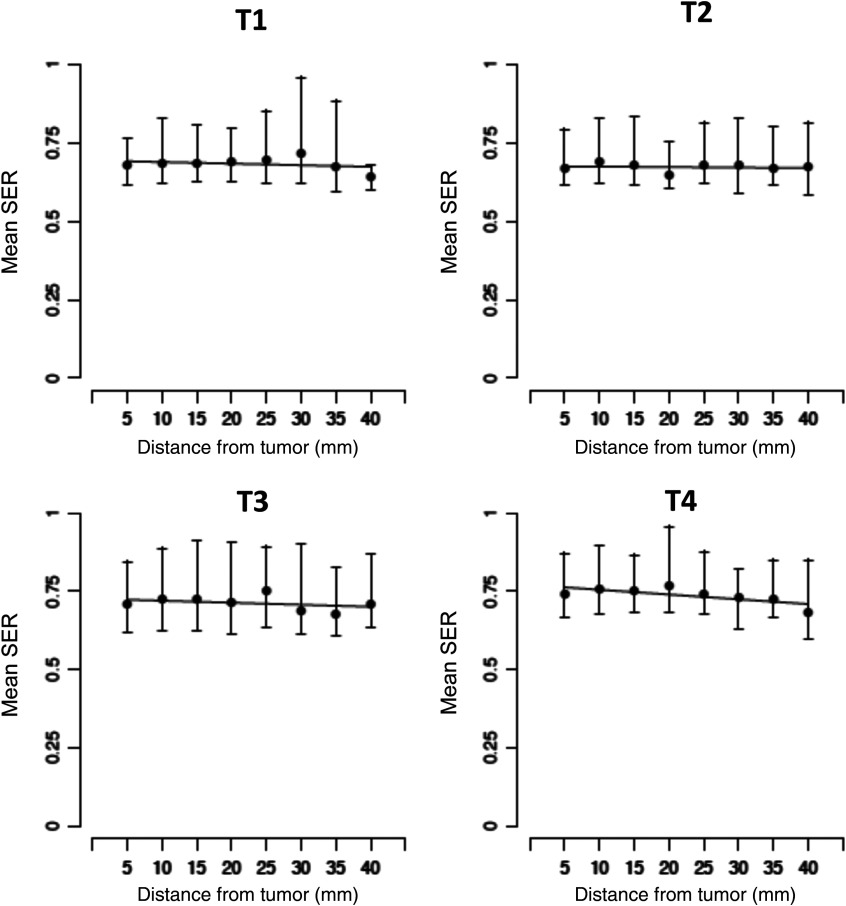
Stromal SER increases with distance from the tumor edge at T1 (P=0.003) but not at other time points.

Using the same mixed effects model as for PE, we observed an increasing trend, rather than a decrease, in mean SER as a function of distance from the tumor edge at the pretreatment time point (T1). Mean SER values increased significantly at T1 only. Here the estimated mean increase per mm was 0.004 (95% CI: 0.001, 0.006; P=0.003) (Fig. 5). In MRIs taken at time points during and after chemotherapy (T2 to T4), changes in SER as a function of distance were not significantly different from zero, Fig. 5.

Similar to PE, we added the recurrence status as a group variable to the mixed effects model with distance to assess differences in SER enhancement patterns in recurrent and nonrecurrent patients. We focused on T1, since that was the only significant time point. The estimated mean increase (95% CI) in the nonrecurrent group was 0.0046 (95% CI: 0.0017, 0.0074; P=0.002) and in the recurrent group was 0.0012 (95% CI: −0.0040, 0.0063, P=0.66).

#### 3.2.3 Global stromal PE and SER and chemotherapy effect

**Fig. 6:**
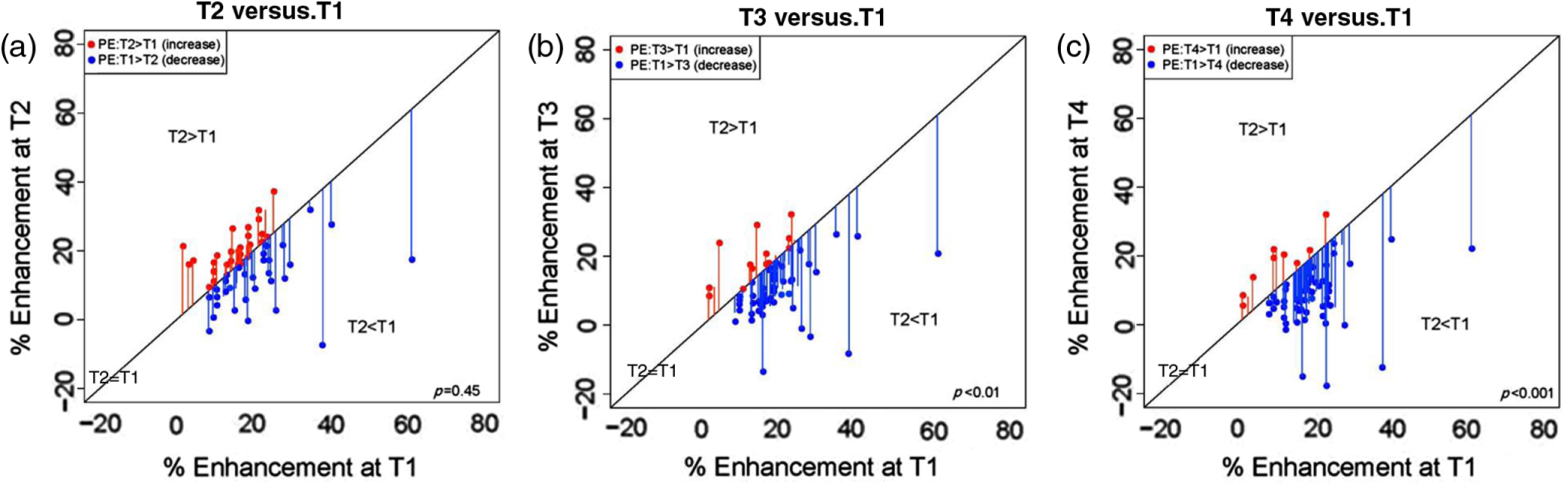
Scatter plot of global PE at each time point for each patient. Global PE decreased significantly with chemotherapy at T3 and T4 (P<0.001) compared to T1.

To determine whether the intrinsic stromal enhancement averaged throughout the breast stroma could be associated with outcomes, we measured global PE at each time point, T1 to T4. Although global PE was not significantly associated with RFS at any time point, we observed a decrease in global PE among the cohort from T1 to T4 (Fig. 6). As shown in the scatter plots, when we examined the PE values at the pretreatment time point (T1), there was no change in PE after the first dose of chemotherapy at T2. However, PE significantly decreased at the interregimen (T3) and pre-surgery (T4) time points relative to pretreatment levels (P<0.001 for each comparison), suggesting that ongoing changes in global PE occurred with increasing exposure to chemotherapy.

Of note, we had previously found that global SER had predictive values among a pilot cohort of patients treated with NAC.^3^^,^^4^ In this paper, a similar analysis using global SER values did not reveal significant differences between T1 and T4, or significant associations between SER and RFS.

### 3.3 Global Stromal PE and SER and Stromal Protein Levels

**Fig. 7:**
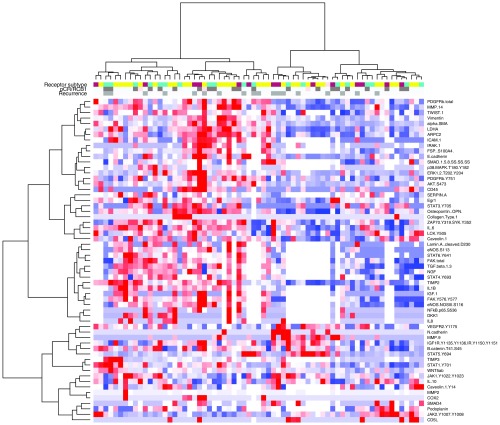
RPPA data at T2. Using unsupervised clustering of RPPA data at T2, two subgroups were identified. (Key for top bar: red = triple negative, yellow=HR+/HER2−, green=HER2+).

Using unsupervised clustering of stromal protein and protein signaling architecture from the RPPA data obtained at T2, two major subgroups were identified (Fig. 7). In the subgroup of 18 patients in which both stromal MR enhancement and protein levels were measured at T2, there were no significant correlations between 10 proteins/phosphoproteins of clinical interest (WNT5ab, VEGFR2 Y1175, Lamin A, cleaved D230, ZAP70 Y319/SYK Y352, PDFGRb, JAK1 Y1022/Y1023, alpha SMA, IL-10, NFkBp65 S536, STAT3 Y705) and measures of stromal MR enhancement (global SER and global PE).

## 4 Discussion

The complex changes associated with cancer-activated stroma reflect dynamic processes of tissue remodeling inherent to tumor progression. Indeed, several features of the cancerous stroma have been found to have prognostic value and have relevance for therapy resistance.^29^ In part, to develop and translate specific stromal properties as predictors of outcomes, more sophisticated tools to assess stromal phenotypes need to be developed. Our previous work was the first to describe low-level quantifiable enhancement characteristics of breast cancer stroma.^3^^–^^5^ As the technical tools have been refined, algorithms to quantify stromal enhancement, to a large degree, have become automated. Using refined techniques, we found that stromal enhancement decreased with exposure to chemotherapy in the 71 patient cohort from I-SPY 1. In addition, we validated previous findings that PE maintained a decreasing pattern with radial distance. These data further validate our previous findings in this cohort from the I-SPY 1 TRIAL.

PE measures the signal enhancement in tissues reflecting the vascular permeability. We previously observed that microvessel density was associated with increased PE in the periphery of tumors, consistent with increased delivery of gadolinium contrast to the extravascular space^5^ and decreased radially with distance from the tumor edge to the surrounding stroma. In the present cohort, we again found a radial dependence of PE from the tumor edge; interestingly, this dependence was maintained through all study time points (T1 to T4). This finding suggests that some degree of vasculature is maintained in tissues proximal to the tumor despite therapy. In the present cohort, due to the limited sample size, we were not able to correlate residual disease or RCB^28^ class with stromal enhancement characteristics. The question of whether stromal enhancement characteristics resolve in patients who achieve pathologic complete response remains interesting and relevant for future investigations.

Given the previously established correlation between microvessel density and increased PE, it was surprising that no correlations in this study were identified between MR enhancement measurements and the protein/phosphoproteins measured. This result may be explained by: (1) the small sample size in which data for both MR and protein measurements were available; (2) differences in MR enhancement attributed to protein/phosphoprotein levels that were not measured in this study; and (3) the average numeric PE and SER descriptors may be limited in describing the regional variation of stromal behaviors. Recent advances in radiomics-based analysis have demonstrated the power of transforming imaging data into multidimensional mineable radiologic features^30^^,^^31^ that are relatable to tissue molecular characteristics and disease prognosis.^32^^–^^34^ Future studies will assess biological differences between stromal specimens with radiomic-based analsysis of MR enhancement patterns in larger cohorts. Radial dependence of protein/phosphoprotein levels will also be investigated.

In addition to measuring radial dependence of PE, we also evaluated the average PE of the entire stroma or global stromal PE at each time point. In the present cohort, global stromal PE decreased with the increasing exposure tochemotherapy from T3 to T4. These findings suggest that the average stromal vascular permeability decreased with chemotherapy, suggesting there are global effects on stromal vasculature that are of unknown significance.

We also measured SER values, which in contrast to PE, reflect washout kinetics of the gadolinium contrast. In our previous studies, we found an association with higher global SER values at posttreatment time points with improved RFS. We did not find this association in the present cohort. However, we observed a small increasing trend in mean SER with distance from the tumor edge at T1. We observed similar associations in our pilot cohort.^4^ While both studies were limited by numbers and events, findings were consistent.

We recognize that the current study population was limited to a cohort of patients enrolled in the I-SPY 1 neoadjuvant trial. Due to relatively small numbers and events, limited conclusions could be drawn from this cohort. However, the findings in this study are consistent with observations made in previous cohorts, namely the radial dependence of PE and SER in the stroma. A new finding is the persistence of the radial dependence of PE despite the treatment with chemotherapy, and the decrease in global PE with the increasing exposure to chemotherapy from T3 to T4.

Enhancement threshold plays a significant role in defining tumor regions and subsequent stromal analyses. In the past, we found that functional tumor volume defined by PE threshold set at 70% has the strongest prediction of RFS^17^^,^^35^ and was used to define the tumor region and subsequent segmentation of the stoma in this study. However, this threshold may be unique to each breast cancer subtype and is a subject of our ongoing research.^36^^,^^37^

We recognize that the current study relied on measurements of average numeric global PE and SER descriptors that may over simplify the behaviors of the tumor microenvironment. However, the current proximity mapping technique uniquely accounts for tissue vascular permeability in all voxels by their location relative to the closest tumor voxel. Ongoing efforts to refine the proximity analysis and integrate methods with radiomic-based analysis may improve the predictive performance for survival. While PE and SER will remain the primary imaging parameters for stromal analysis in DCE-MRI, the proximity mapping methodology can also be extended to diffusion-weighted imaging. Other descriptors such as apparent diffusion coefficient^38^ or fractional anisotropy (FA)^39^ measurements that depict tumor cellularity and water mobility may provide additional important information for breast stromal characterization.
